# Pain reduction in fibromyalgia syndrome through pairing transcranial direct current stimulation and mindfulness meditation: A randomized, double-blinded, sham-controlled pilot clinical trial

**DOI:** 10.3389/fmed.2022.908133

**Published:** 2022-10-12

**Authors:** Perianen Ramasawmy, Sarah Khalid, Frank Petzke, Andrea Antal

**Affiliations:** ^1^Department of Neurology, University Medical Center Göttingen, Georg-August University, Göttingen, Germany; ^2^Department of Anesthesiology, University Medical Center Göttingen, Georg-August University, Göttingen, Germany

**Keywords:** transcranial direct current stimulation (tDCS), fibromyalgia (FMS), meditation, mindfulness, brain stimulation

## Abstract

**Background:**

This double-blinded, randomized and sham-controlled pilot clinical trial aimed to investigate the preliminary clinical efficacy and feasibility of combining mindfulness meditation (MM) and transcranial direct current stimulation (tDCS) for pain and associated symptoms in patients with fibromyalgia syndrome (FMS).

**Methods:**

Included FMS patients (age: 33 to 70) were randomized to three different groups to receive either ten daily sessions of anodal tDCS over the left primary motor cortex paired with MM for 20 min (active + MM, n = 10), sham tDCS combined with MM (sham + MM, *n* = 10) or no intervention (NoT, *n* = 10). Patients in the bimodal therapy groups received a week of training in MM prior to the stimulation. Participants reported pain intensity, the primary outcome, by filling in a pain diary daily throughout the whole study. They were also evaluated for quality of life, pressure pain sensitivity, psychological wellbeing, sleep quality and sleep quantity. Assessments were performed at three time points (baseline, immediately after treatment and one-month follow-up).

**Results:**

Participants in the active + MM group did not exhibit reduced pain intensity following the bimodal therapy compared to controls. Patients in active group demonstrated clinically meaningful and significantly higher quality of life following the therapeutic intervention than other groups. There was no significant difference among groups regarding pressure pain sensitivity, sleep parameters and psychological scales. The combined treatment was well tolerated among participants, with no serious adverse effects.

**Conclusion:**

This study was the first to pair these two effective non-pharmacological therapies for pain management in FMS. In the light of an underpowered sample size, repetitive anodal tDCS combined with MM did not improve pain or FMS-associated symptoms. However, patients in the active + MM group reported higher quality of life than the control groups. Studies with more participants and longer follow-ups are required to confirm our findings.

**Clinical trial registration:**

[www.drks.de], identifier [DRKS00023490].

## Introduction

Fibromyalgia syndrome (FMS) is a heterogeneous primary pain condition, characterized by persistent and widespread non-inflammatory musculoskeletal chronic pain. FMS has an incidence rate of 2-5% in the worldwide population, with the majority of the patients consisting of women (1). FMS carries a large burden on patients owing to the cutbacks in daily life caused by chronic pain and associated symptoms such as sleep disturbances, fatigue, cognitive impairments and psychological problems (2, 3). The most recent European League against Rheumatism guidelines recommend that once FMS diagnosis has been made, the primary focus should be on non-pharmacological therapies rather than treatment with medication (4, 5). Thus, there is great interest in developing innovative non-pharmacological therapies to manage FMS pain and related symptoms.

Two such treatments that have shown pain reduction in FMS patients are transcranial direct current stimulation (tDCS) and mindfulness meditation (MM). tDCS comprises the application of a weak direct electric current to the scalp delivered *via* surface electrodes for effective, safe and non-invasive brain stimulation in humans (6, 7). Despite the small induced subthreshold change in the membrane potential (0.2-0.5 mV), tDCS delivered at an intensity of 1-2 mA induces excitability changes at both local and network levels (8, 9). The two most studied common stimulation targets are the M1 and the dorsolateral prefrontal cortex (DLPFC). Many studies have shown the therapeutic benefits of anodal M1 tDCS in FMS in terms of clinical pain, quality of life and psychological wellbeing (10–14), with effects lasting up to two months following stimulation (15, 16). Nevertheless, Foerster et al. (17) failed to demonstrate significant pain reduction in patients with FMS after 5 days of anodal tDCS of M1 compared to sham stimulation (17). Prefrontal anodal tDCS has been associated with cognitive improvements and the emotional aspects of pain reduction (18). However, anodal stimulation of M1 demonstrated better analgesic effects over DLPFC stimulation (18). Recent evidence-based guidelines for the therapeutic use of tDCS recommended ten daily 20-minute sessions of anodal tDCS at the left primary motor cortex (M1) applied at a current of 2 mA for probable efficacy in FMS pain (19, 20). Lately, Samartin-Veiga et al. (21) showed that a fifteen sessions of 20-min anodal tDCS at 2 mA over either the left M1 or left DLPFC or left operculo-insular cortex in FMS patients showed similar improvement in clinical pain and associated symptoms to sham group, challenging the efficacy of tDCS in FMS treatment (21).

Mindfulness meditation is a cognitive training practice, which fosters the ‘detached, non-judgmental and non-elaborative’ awareness of the present moment. Over the decades, there has been a significant surge in the scientific evidence supporting the effectiveness of MM in FMS and other chronic pain conditions (22–26). Parra-Delgado and Latorre-Postigo (27) found a reduction in disease impact and in depressive symptoms of FMS patients following an eight-week program of mindfulness cognitive therapy (27). In a study by Cash et al. (28), an eight-week mindfulness-based stress reduction program ameliorated stress, sleep disturbances and severity of symptoms in FMS without significant differences in pain intensity ratings compared to a wait-list control group (28). A recent trial showed that an 8-week meditation awareness training program ameliorated pain and FMS-related symptoms to a greater extent than an equal period of cognitive based therapy (29).

The analgesic effects of anodal tDCS in chronic pain patients have been shown to be boosted when it is combined with other non-pharmacological interventions (30, 31). Concurrent application of tDCS and MM in adults with knee osteoarthritis with no prior meditation training showed amelioration of pain and symptoms compared to a control group receiving sham tDCS paired with sham MM (32). Our current study, to our best knowledge, is the first to test the potential therapeutic effects of combining these two non-pharmacological therapies in FMS patients. The rationale behind this combination lies in potential additive or even synergistic effects on pain modulation by tDCS and MM, which might lead to more positive and longer lasting clinical outcomes.

The primary aim was to test the feasibility and efficacy of two weeks of the tDCS and MM intervention in pain reduction in FMS patients following a one-week training in meditation in a pilot clinical trial. We hypothesized that the participants in the active stimulation group pairing anodal tDCS at left M1 and MM will report greater pain relief than those who received a combination of MM and sham stimulation and those who received no therapeutic intervention. The no treatment group served as a control for symptom variability in FMS patients. We also examined the impact of the bimodal therapy on pain sensitivity, quality of life, psychological wellbeing and sleep.

## Materials and methods

All participants were informed prior to inclusion about the protocols in detail. Written informed consent was signed before participation. This pilot clinical trial was approved by the Ethics Committee of the University of Göttingen under the registration number 33/8/20 and is registered at www.drks.de (DRKS00023490). All experimental procedures conformed to the ethical standards of the 1964 Declaration of Helsinki and its revisions.

### Participants

**FIGURE 1:**
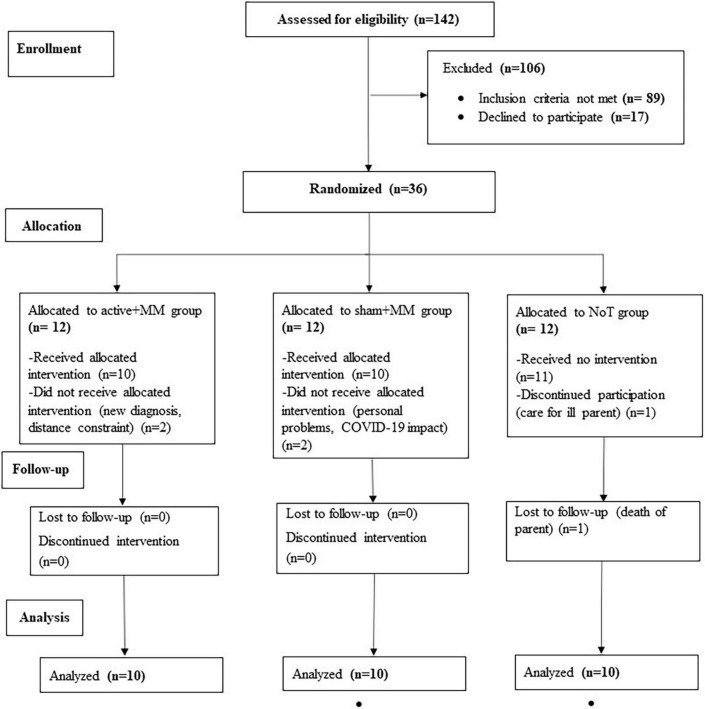
CONSORT diagram of the clinical trial. The flow chart shows the number of FM patients enrolled, allocated to each group, completed the study and included in the final analysis.

Patients diagnosed with FMS were recruited through the outpatient pain clinic of the Department of Anesthesiology, University Medical Centre, Göttingen, reports in local newspapers, advertisements in the hospital, social media and patient support groups for fibromyalgia and rheumatological conditions. Individuals from 30 to 70 years old were considered suitable to participate if they fulfilled the preliminary American College of Rheumatology Diagnostic Criteria 2010 for FMS with a widespread pain index ≥ 7 and a symptom severity score ≥ 5 (2). Further inclusion criteria were stable chronic pain for at least 6 months prior to participation, stable analgesic or psychotropic medications for ≥ 4 weeks prior to the study and no new treatment approaches over the preceding 4 months. Participants were excluded if they (a) were undergoing treatment with strong opioids or taking more than three medications for FMS treatment; (b) were being treated with carbamazepine, benzodiazepines, phenytoin, gabapentin, pregabalin; calcium channel antagonists (e.g., flunarizine), NMDA receptor antagonists (e.g., dextromethorphan, memantine) or anticholinergics; (c) had any major or unstable medical or psychiatric disorder; (d) were diagnosed with severe or uncontrolled comorbid rheumatic disease; (e) had active alcohol or drug addiction; (f) were pregnant or breastfeeding; (g) had a history of unexplained or repeated loss of consciousness; (h) had implanted metallic devices in head, neck or chest; (i) had participated in another scientific or clinical study within 12 weeks prior to study inclusion; or (j) had had any planned surgery or hospitalization which might have conflicted with or influenced their participation in our study. Participants were allowed to continue ongoing psychotherapy (up to 2x/month) and/or physiotherapy (up to 2x/week). Alternative therapies (muscle relaxation and acupuncture) and homeopathic remedies were allowed, given they had been stabilized at least four weeks prior to inclusion in the study, and the therapy was pursued at similar intensity during the study. Out of 142 patients screened, 36 participants were included, of whom 6 dropped out (Figure 1). Finally, 30 patients (age range 33-70 years; 2 males) completed the study. None of the participants were ardent meditation practitioners and they were all considered novice to the regular practice of MM.

The study was a monocenter, parallel, randomized, placebo-controlled clinical trial to test the efficacy of ten daily 20-minute sessions of anodal tDCS over the left M1 paired with MM over two weeks (Monday to Friday) in patients with FMS. The included participants were randomized to 3 intervention groups: active + MM (n = 10), which received anodal tDCS paired with MM; sham + MM (n = 10), which received sham tDCS combined with MM; and NoT (n = 10), which received neither tDCS nor MM. Randomization was performed after inclusion using the randomizer function from *GraphPad 2021* software. The patients in sham + MM or NoT were invited to receive the active tDCS treatment following the completion of the study.

The study was double blind, as both patient and experimenter were unaware of the tDCS protocol (sham or active). Blinding was maintained and executed by the study leader, who programmed the stimulators and was not involved in experiments. However, there was no blinding with regard to the NoT group. Pressure algometry was performed by the blinded experimenters, unblinding occurred after completion of the study.

**FIGURE 2:**
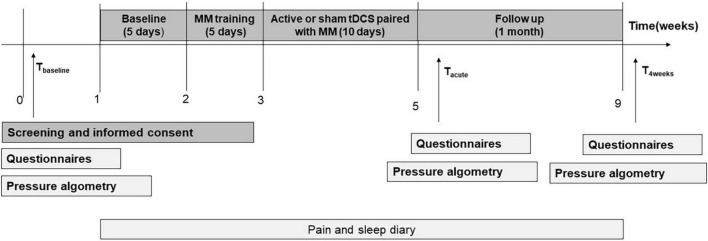
Study design for the active + MM and sham + MM treatment groups.

For the participants in the active + MM or sham + MM groups, the study had four phases (a) 1 week baseline period; (b) 1 week (Monday to Friday) of daily MM training; (c) 2 weeks of daily bimodal therapy intervention (Monday to Friday) groups and (d) one month of follow-up period, as illustrated in Figure 2.

The NoT group did not receive any meditation training or bimodal therapy. Participants in the NoT group also filled in the pain and sleep diary daily over the whole study period of 9 weeks and all other measurements likewise at the baseline visit (T_baseline_). They did not have any further scheduled appointment at the hospital apart from T_acute_ and T_4weeks_ evaluations limited to questionnaire data and pressure algometry and taken at similar time points as for the groups receiving the therapeutic interventions.

### Outcomes

Pain intensity, sleep quality and sleep quantity were rated daily in a diary by the participants for the whole duration of the study. All other outcome variables were measured one week before the start of MM training (T_baseline_), within 5 days (T_acute_) and after 4 weeks (T_4weeks_) following the last stimulation session. The level of mindfulness was measured on the first and the last day of the MM training.

#### Primary outcome

The Numerical Rating Scale (NRS) was used to assess pain intensity, as self-reported by the patient. This 11-point scale comprising integers ranged from 0 to 10; 0 meaning no pain and 10 indicating the worst imaginable pain. Patients rated their pain on the NRS twice a day (after waking up in the morning and before going to sleep in the evening) throughout the whole study. The pain scores were averaged to give one NRS mean value for each study week. When a patient missed one NRS value on a particular day, the missing value was given the score for the other time of the same day. Some patients reported their pain as a range of values (e.g., 7-8) and the NRS score was taken as the mean (i.e., 7.5). The reproducibility and validity of the NRS has been shown, making it a reliable measure for pain assessment as a clinical outcome in many studies (33–35). At least 20% reduction on the mean NRS was considered a clinically relevant improvement of pain (36, 37).

#### Secondary outcomes

##### Quality of life

Quality of life and functional capacity of patients was assessed using the German version of the Fibromyalgia Impact Questionnaire (FIQ; Cronbach’s α = 0.92) (38). The latter is an assessment and evaluation tool used to measure the status, progress and outcomes of FMS patients (39). The FIQ is a 10-item questionnaire, where the first item is related to overall physical function, such as the ability to go shopping, to prepare food and to drive a car. The patients’ answers are rated on a 4-point Likert scale (0: always and 3: never) for the ability to do different tasks. Item 2 asks the patient to encircle the number of days they felt well and item 3 the number of days they could not go to work (excluding home office) owing to FMS symptoms. The last seven items require the patient to rate their fatigue, pain, tiredness, work difficulty, stiffness, depression and nervousness on a 10 cm horizontal linear scale. The total score is expressed as the sum of all the items (range, 0 to 100) after correcting the first three items to a maximum score of 10, with higher scores indicating poorer quality of life (40). At least 14% reduction on the FIQ score is considered a clinically relevant improvement in quality of life (41).

##### Pressure pain threshold

Pressure pain threshold (PPT) is a reliable and reproducible measure of pain sensitivity for FMS (42, 43). The pain sensitivity test was performed using a hand-held pressure algometer (Algometer O, Somedic Sales, Hörby, Sweden) perpendicular to the skin through a 1-cm^2^ probe bilaterally at 8 sites: bilateral mid-trapezius, lateral epicondyle, mid-thigh and tibialis anterior muscles. The same experimenter performed the PPT at the three time points for one patient. The pressure was gradually increased by 50 kPa/s until the patient said ‘Stop’ or when their perception first changed from pressure to pain. The maximal stimulation pressure was 1,000 kPa to avoid tissue damage. We administered the pressure algometer in the same order for all patients- right tibialis, right midthigh, right epicondyle, right mid-trapezius, left mid-trapezius, left epicondyle, left mid-thigh and left tibialis. For each site, PPT was measured twice with at least a 30s interval (44) and the mean of the two recordings was calculated for further analysis. If the difference of the two PPTs at a particular site was greater than 100 kPa, a third measurement was obtained and the mean of the three values calculated. The experiment was conducted in a quiet room without any disturbances. The patient sat comfortably in a chair, with their feet slightly separated.

##### Psychological wellbeing

The validated German version of the Depression, Anxiety and Stress Scale- 21 Items (DASS), comprising three self-report subscales, was used to evaluate depression (Cronbach’s α ≥ 0.91), anxiety (Cronbach’s α = 0.78–0.82) and stress (Cronbach’s α = 0.81–0.89) of the participants (45, 46). Each subscale of the DASS comprises seven questions, rated from 0 (did not apply to me at all) to 3 (applied very much or most of the time). Each measure yields a total subscale score, which is the sum of the scores of the seven items multiplied by 2. Depending on the scores, the participant was allocated to one of five categories referring to the severity of the psychological condition: normal, mild, moderate, severe and extremely severe (45).

##### Sleep quality and quantity

The NRS was used to assess sleep quality, as reported by the patient. This 11-point scale comprising integers ranged from 0 to 10; 0 meaning no restful sleep and 10 indicating the maximum restful sleep. Participants rated their sleep quality of the preceding night every morning just after waking up in the diary. The scores were averaged to give one NRS mean value for each week.

The number of hours the participant slept the previous night was recorded in the diary by the patient every morning just after waking up. The sleep quality scores were averaged to give one mean value for each week.

##### Mindfulness level

The German version of the 14-item short-scale of the Freiburg Mindfulness Inventory (FMI) served as a monitor whether the patients were able to engage in mindfulness practice and learn how to meditate (47). The FMI is a psychometrically validated tool with high sensitivity and internal consistency (Cronbach’s α = 0.84) (47, 48). Scores range from 14 to 64, with higher values representing enhanced mindfulness skills.

### Interventions

#### Brief mindfulness meditation training (5 days)

Participants in the active + MM (*n* = 10) and sham + MM (*n* = 10) groups underwent the 5-day long MM training. The MM training intervention was the same for both groups. The brief MM training and meditation protocols were designed and instructed by a meditation teacher who had more than 20 years of training in teaching mindfulness. The training was designed based on the guide for mindfulness practice as defined in Kabat-Zinn (22). They sat on a comfortable chair in a relaxed, yet alert position, except two patients who preferred to lay down on a mat owing to their inability to sit still for a longer time. In every meditation session, participants meditated to a pre-recorded guided meditation audio. The meditation sessions during both training and stimulation weeks included a 5-min body scan meditation exercise and a 20-min MM exercise. The body scan emphasized focus on the present-moment awareness of different sensations and feelings within various body parts, and ended with instructing the participant to feel all parts of the body as a whole (49). In our study, the MM exercise involved developing focused attention on the breathing, which was the object of awareness. The attention was then expanded to include a non-judgmental, non-attached and more open observation of any sensory, cognitive or emotional experiences (22).

Thus, on the first two days, the 20-min MM exercise focused on developing awareness of the breathing by using only bodily sensations as guidance whereas during the rest of the training week, the 20-min exercise emphasized bringing attention to the breath while using both body and mental objects (thoughts and feelings) as tools. Following each meditation training session, we encouraged patients to ask questions and report difficulties experienced during the meditation. On the last two days of MM training, the electrodes, the cables and the rubber bandages were placed on the head of the subject (without stimulation) to mimic the conditions for the combined MM and tDCS intervention. Additionally, prior to every meditation session during both the training week and bimodal therapy weeks, a 5-min nature sound audio – consisting of birds chirping, river flowing and forest sounds – was played to relax the participants.

Previous studies applying mindfulness training in chronic pain often use 8-week long mindfulness interventions (27–29). Zeidan et al. (50) showed that a four-session MM training significantly increased mindfulness level assessed with FMI in meditation-naïve university students who completed the training versus controls (50). It has also been shown that a 3-day (1-hour total) MM training reduced heart rate and negative mood in the meditating group compared to control groups (51). However, both these studies were conducted in healthy young university students. Our study was the first to investigate the effects of a brief 5-session MM training on mindfulness level, assessed with the FMI, in FM patients.

In addition to the FMI each participant was asked after the last two MM training sessions, whether “they had the feeling that they were truly meditating” (adapted from (51)) to assess whether the participants thought they had learned the meditation technique). They were asked to respond with either ‘Yes’ or ‘No’.

#### Transcranial direct current stimulation

A constant direct current was applied daily for 20 minutes over two weeks (Monday to Friday) *via* a pair of surface electrodes connected to a NeuroConn DC-Stimulator Plus (NeuroConn, Illmenau, Germany). The anode (4 cm x 4 cm; 16 cm^2^) was placed over the left M1, which was defined as the point 5 cm from C_z_ in the direction of the left preauricular notch measured with a measuring tape. The anode size was large; therefore, the area it covered encompassed a broad area of the motor cortex. The reference electrode (5 cm x 10 cm; 50 cm^2^) was placed over the right supraorbital frontal area. The dimensions of the electrodes were based on a previous study (52). To fix the electrodes and to reduce electrical resistance between scalp and electrode, AC Cream electrode paste (Spes Medica S.r.l., Genova, Italy) was used. Rubber bandages were used to hold the position of the electrodes during stimulation. Each participant received the daily stimulation at the same time of the day and it was ensured that the direction of the cables were the same for all patients and all stimulation sessions (53).

For the active anodal tDCS of M1, a constant current of 2 mA with 15 s ramp up at the beginning and 15 s ramp down at the end was applied for 20 min. Stimulation with 2 mA intensity has been shown to be safe for use in both healthy individuals (54) and chronic pain patients (52, 55). For the sham stimulation, the position of electrodes was identical to active tDCS. However, the current was switched off automatically after the first 30 s of stimulation. The reliability of this sham technique has been previously validated (52, 56) and it has been shown to be indistinguishable from active stimulation (57).

We noted the resistance value from the stimulator at 5 minutes following the start of the stimulation and it was ensured that the resistance was below 10 kΩ (58). The mean resistance across all patients and stimulation sessions was 4.5 ± 1.5 kΩ (mean ± standard deviation; min = 2.0 kΩ, max = 7.5 kΩ). Following every stimulation session, patients reported the presence of any side or adverse effects of the stimulation: uncomfortable skin sensation under the electrodes (tingling, warmth), headache, vertigo, tiredness and nervousness. Skin redness under the stimulation electrodes was also noted.

To estimate the success of blinding in the study, we implemented the end-of-study guess approach, which is a commonly used method in tDCS studies (59–61). Participants were informed that they would either receive active or sham tDCS, but were blinded to the stimulation type. After the last stimulation session, they were asked to guess whether they received the active or sham stimulation. We assessed blinding success using the Chi-squared test to investigate whether the guessing of the type of stimulation received was different from the chance level (50%). The correct sham guesses (7 out of 20 patients, 35%) were at the same level as chance (χ^2^ = 1.80, p = 0.180), suggesting a successful blinding.

#### Concurrent mindfulness meditation and transcranial direct current stimulation therapy

During the two treatment weeks, each session started with 5 minutes of body scan exercise followed by 20 minutes of guided MM identical to the audios used for the last three days of meditation training. The stimulator was switched on immediately after the body scan and the start of stimulation was paired with the start of the 20-minute meditation. We included the body scan exercise to ensure that participants were actually already meditating at the onset of stimulation.

### Statistical analysis

All participants who completed the intervention phase attended both follow-up visits to the clinic. All statistical analyses were performed using *IBM SPSS 28.0* and graphs were plotted in *GraphPad Prism 9*. Normality of raw data or residuals was verified with the Shapiro-Wilk test and visually with normality plots. Non-parametric tests were used where parametric assumptions for tests were violated or data could not be transformed to meet the assumptions. For mixed model ANOVA (MANOVA) analyses, the sphericity assumption for the repeated factor was checked with Mauchly’s test and Greenhouse- Geisser corrections were applied to the degrees of freedom for deviations from sphericity. The homoscedasticity assumption for independent groups was assessed with Levene’s test and the variables were corrected to baseline if the assumption was violated. Effect size was computed as eta squared η^2^ and was interpreted based on Cohen’s benchmark categorization (small, η^2^ = 0.01; medium, η^2^ = 0.06 and large, η^2^ = 0.14) (62). Data were expressed as mean and SEM both for analysis and in graphs. Statistical significance was set at *p* < 0.05.

To test the effects of the bimodal therapy on the primary outcome variable - NRS pain intensity - we used a two-way MANOVA, with WEEK (baseline, MM training week, first week of stimulation (Stim1), second week of stimulation (Stim2), four follow-up weeks (PostStim1, PostStim2, Poststim3, PostStim4)) as the repeated factor and GROUP (active + MM, sham + MM, NoT) as the independent factor. *Post hoc* analyses were conducted using Least Significant Difference (LSD) test following Bonferroni correction to test how pain intensity changes over time compared to baseline measure.

The data for the sleep quantity and quality were analyzed in the same way as for NRS pain intensity. For the FIQ and PPT measures, two-way MANOVAs were run with the respective outcome variable as the dependent variable, with TIME (T_baseline_, T_acute_, T_4weeks_) and GROUP as repeated and independent factors, respectively. For each DASS subscale, To test the effects of the MM training on mindfulness level (*n* = 20), a two-way MANOVA was conducted for FMI scores, with DAY (day 1 and day 5 of MM training week) and MM GROUP (active + MM, sham + MM) as factors.

To test the clinical relevance of pain improvement over time across groups, the number of patients in each group exhibiting ≥ 20% decrease in NRS compared to baseline was counted. A three-way contingency table analysis for proportions was carried out with GROUP, follow-up weeks and whether they showed sufficient decrease in NRS as categories. A similar analysis was done for the clinical relevance of quality of life. However, the categories included GROUP, TIME (T_acute_, T_4weeks_) and whether they showed ≥ 14% reduction in FIQ scores. The Fisher-Freeman-Halton Exact test was used for both analyses owing to violation of Chi-squared test assumptions.

We also investigated the correlation between pain intensity at PostStim1 and PostStim4 and FIQ scores at T_acute_ and T_4weeks_, respectively, across all groups. Both variables were corrected to baseline.

## Results

Of the 30 participants who completed the study, two were males. The mean age of the total sample was 53.60 ± 1.73, mean symptom severity was 8.66 ± 0.36 and mean widespread pain index was 14.2 ± 0.53. The groups were not age-matched. The baseline demographics and clinical characteristics are available in Table 1. Table 2 presents the individual patient clinical and demographic variables including their ongoing FMS treatment with medication, physiotherapy or psychotherapy.

**TABLE 1:** Baseline demographic and clinical characteristics among groups.

Variables, M ± SD (min to max)	Total (*N* = 30)	NoT group (*n* = 10)	Sham + MM group (*n* = 10)	Active + MM group (*n* = 10)	*P*-value
Age, years	53.60 ± 9.48 (33 to 70)	52.80 ± 8.47 (41 to 64)	48.20 ± 2.70 (33 to 59)	59.70 ± 8.37 (47 to 70)	0.02
*Sex, n (%)*					0.31*
Female	28 (93.33)	10 (100)	8 (80)	10 (100)	
Male	2 (6.67)	0 (0)	2 (20)	0 (0)	
BMI, kg/m^2^	27.5 ± 5.48 (23 to 32)	29.05 ± 5.96 (22 to 41)	26.28 ± 3.57 (20 to 32)	28.95 ± 6.47 (18 to 42)	0.99
Right handed, n (%)	26 (86.67)	8 (80)	9 (90)	9 (90)	1.00*
SS	8.66 ± 1.97 (5 to 12)	8.35 ± 2.06 (6 to 12)	9.1 ± 1.97 (6 to 12)	8.30 ± 2.00 (5 to 12)	0.49
WPI	14.2 ± 2.91 (9 to 18)	14.60 ± 2.84 (10 to 18)	12.80 ± 3.05 (9 to 18)	15.20 ± 2.53 (11 to 18)	1.97
**Baseline measures**					
NRS pain intensity	5.57 ± 1.57 (1.36 to 8.79)	5.32 ± 1.60 (1.36 to 7.00)	5.65 ± 1.60 (3.04 to 8.37)	5.71 ± 1.66 (2.86 to 8.79)	0.89**
PPT, kPa	196 ± 60 (103 to 387)	188 ± 44 (133 to 274)	208 ± 86 (103 to 387)	190 ± 46 (114 to 259)	0.76
FIQ	51.5 ± 14.4 (19.6 to 84.4)	49.7 ± 7.8 (32.9 to 60.7)	50.9 ± 21.3 (19.6 to 84.4)	54.2 ± 12.0 (39.9 to 80.2)	0.78
*DASS-21 questionnaire*					
Depression score	5.5 ± 4.0 (0 to 16)	5.3 ± 2.6 (2 to 10)	5.7 ± 5.5 (0 to 16)	5.6 ± 3.6 (1 to 14)	0.98
Anxiety score	3.9 ± 3.0 (0 to 12)	4.5 ± 3.7 (1 to 12)	4.0 ± 2.9 (0 to 8)	3.2 ± 2.3 (0 to 6)	0.84**
Stress score	8.2 ± 3.8 (2 to 16)	8.5 ± 2.7 (5 to 13)	8.3 ± 5.0 (2 to 16)	7.9 ± 3.7 (2 to 15)	0.94
NRS sleep quality	4.7 ± 1.5 (1.6 to 8.6)	5.0 ± 1.5 (2.3 to 7.0)	4.0 ± 1.0 (1.6 to 5.6)	5.0 ± 1.7 (2.0 to 8.6)	0.23
Sleep quantity, hours	7.1 ± 1.3 (4.3 to 9.9)	7.5 ± 1.5 (4.7 to 9.9)	6.8 ± 1.2 (4.9 to 8.1)	7.0 ± 1.4 (4.3 to 8.9)	0.52
FMI (n = 20)	37 ± 5 (25 to 46)		35 ± 6 (25 to 40)	38 ± 4 (31 to 46)	0.20***

M , mean; SD , standard deviation; BMI , body mass index; SS , symptom severity (range, 0 to 12); WPI , widespread pain index (range, 0 to 19); NRS , numerical rating scale; PPT , pressure pain threshold; FIQ , fibromyalgia impact questionnaire; DASS-21 , depression, anxiety and stress scale; FMI , Freiburg mindfulness inventory. Categorical data was analyzed using Fisher-Freeman-Halton exact test (*) and the rest was analyzed using either one-way ANOVA or Kruskal Wallis test (**) depending on whether parametric assumptions were met. FMI data was analyzed using an unpaired t-test (***).

**TABLE 2:** Individual participant demographic and clinical parameters.

Patient	Group	Sex	Age (years)	FMS related medication	Physiotherapy (x/week)	Psychotherapy (x/month)
1	Sham + MM	F	50	NSAID awr	0	1
2	Active + MM	F	70	Adalimumab Metamizole awr	1	0
3	Sham + MM	F	51	Trimipramine Novalgin awr CBD	1	0
4	Active + MM	F	68	Tilidine	2	1
5	No treatment	F	64	Trimipramine awr	2	0
6	Sham + MM	F	38	—	1	2
7	No treatment	F	56	—	1	0
8	Active + MM	F	57	—	0	2
9	Active + MM	F	68	Metamizole awr Diclofenac awr	0	0
10	No treatment	F	58	Celecoxib awr	1	0
11	No treatment	F	41	Duloxetine Opipramol	1-2	0
12	Sham + MM	F	58	Amitriptyline Mirtazapine	0	0
13	No treatment	F	45	Ramipril	2	0
14	Active + MM	F	68	—	0	0
15	Sham + MM	M	33	NSAID awr	0	0
16	No treatment	F	53	Novalgin awr	2	4
17	Sham + MM	M	59	Etoricoxib	1	0
18	Active + MM	F	55	NSAID awr	0	0
19	Active + MM	F	50	Paracetamol awr	0	0
20	Active + MM	F	47	NSAID awr Paracetamol awr	0	0
21	Active + MM	F	60	Amitriptyline	1	0
22	Sham + MM	F	49	NSAID awr	2	1
23	Sham + MM	F	48	—	0	0
24	Active + MM	F	54	Pregabalin	1	0
25	Sham + MM	F	55	NSAID awr Tilidine (by very strong pain)	1	2
26	Sham + MM	F	41	Novalgin awr	0	2
27	No treatment	F	64	—	0	0
28	No treatment	F	48	Etoricoxib awr Fluoxetine	1	0.5
29	No treatment	F	57	NSAID awr Pregabalin	0.5	1
30	No treatment	F	42	NSAID awr	0	0

F , female; M , male; awr , as and when required; NSAID , non-steroidal anti-inflammatory drugs; CBD , cannabidiol; — , none.

### Primary outcome: Numerical rating scale for pain intensity

**FIGURE 3:**
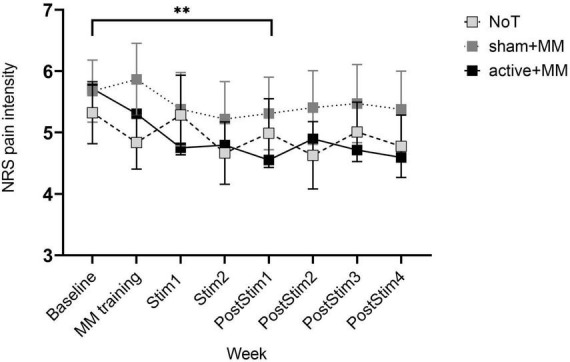
Pain intensity scores as cataloged by NRS during and after concurrent meditation and tDCS intervention. Bars show standard error of mean. ^**^ *p* < 0.01.

With regard to the NRS pain intensity, the mixed model ANOVA failed to show any significant differences among the groups (F_GG_ (2, 27) = 0.36, *p* = 0.703) and any interaction between GROUP and TIME (F_GG_ (0.84, 114.71) = 0.87, *p* = 0.553). However, there was a significant medium main effect of WEEK (F_GG_ (4.25, 114.71) = 2.85, *p* = 0.025, η^2^ = 0.095) (Figure 1). By *post hoc* analysis, the mean NRS at PostStim1 ($\bar{\text{x}}$ = 5.57 ± 0.29) was lower than baseline ($\bar{\text{x}}$ = 4.95 ± 0.32) (*p* = 0.003), with no other significant pairwise comparisons (Figure 3). This shows an acute reduction in pain intensity following the combined therapy phase compared to baseline in all intervention groups.

**FIGURE 4:**
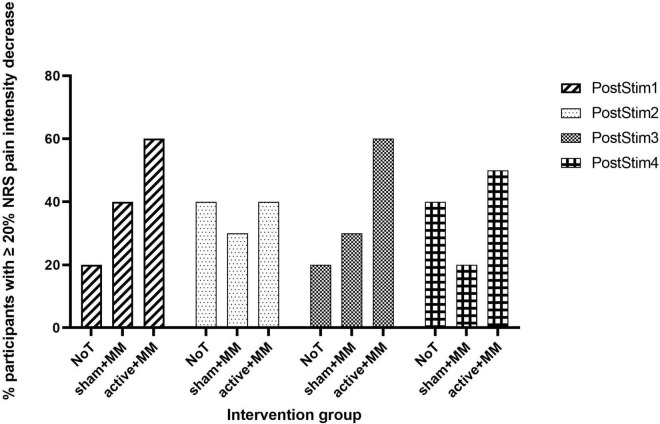
Clinically meaningful pain relief. Bar chart illustrating the percentage of participants who reported ≥ 20% reduction in NRS pain intensity at each follow-up post stimulation week compared to baseline in the NoT, sham + MM and active + MM groups.

Concerning the clinical relevance of the pain reduction, we did not find any significant association between proportion of patients with ≥ 20% decrease in NRS and group at all follow-up weeks (PostStim1: *p* = 0.248; PostStim2: *p* = 1.00; PostStim3: *p* = 0.249; PostStim4: *p* = 0.510). Nevertheless, visual representation of the percentage of patients with clinically relevant pain decrease across group and time illustrated the following: (1) the percentage of patients in active + MM group was always higher than sham + MM group at each follow-up week and (2) the percentage of patients in NoT group fluctuated over time, demonstrating no consistent pattern (Figure 4).

**FIGURE 5:**
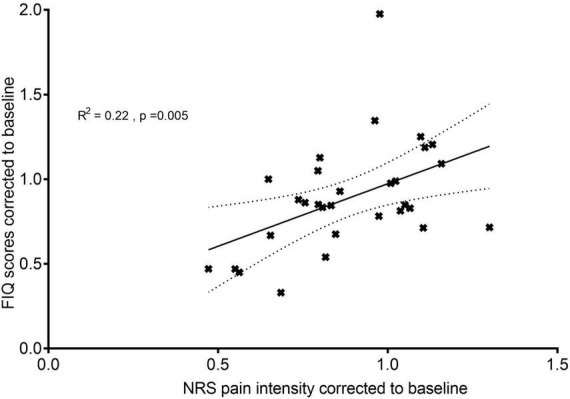
Relationship between quality of life and pain intensity: acute effects. A correlation analysis between FIQ scores at T_acute_ corrected to T_baseline_ and NRS pain intensity at PostStim1 corrected to baseline. *N* = 30, all patients were included for this analysis. The fitted line is the plotted linear regression as mean with standard error of the mean.

Acute (PostStim1) baseline-corrected pain intensity correlated positively with acute baseline-corrected FIQ scores (*r* = 0.466, *p* = 0.005, one-tailed Pearson correlation), which accounted for 22% of the total variation in the data (Figure 5). No significant association was found between the two variables at T_4weeks_ (*r* = 0.255, *p* = 0.087, one-tailed Pearson correlation).

### Secondary outcomes

#### Quality of life: FIQ

**FIGURE 6:**
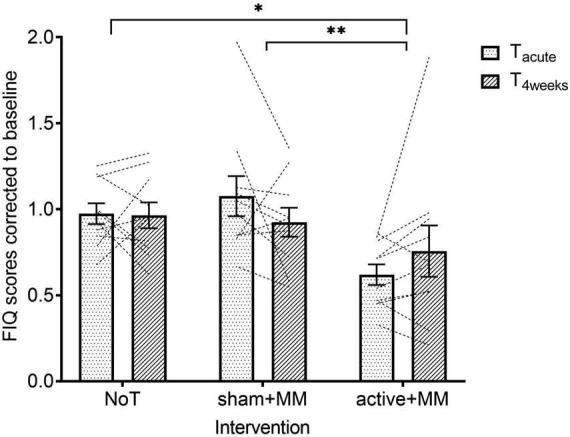
Effects of combining MM and tDCS on quality of life. Graph shows how the baseline corrected mean FIQ scores change over time among the NoT, sham + MM and active + MM. Bars show standard error of mean. * *p* < 0.05. ^**^ *p* < 0.01.

For the FIQ scores, the raw data had to be corrected to baseline to meet the homoscedasticity assumption. Quality of life showed a significant main large effect for GROUP (F (1, 27) = 4.34, *p* = 0.023, η^2^ = 0.243) but not TIME (F (1, 27) = 0.021, *p* = 0.885) and TIME-GROUP interaction (F (2, 27) = 2.10, *p* = 0.142). Following pairwise comparisons with Sidak *post hoc* test, MM + active group ($\bar{\text{x}}$ = 0.688 ± 0.080) showed lower FIQ scores than both sham + MM ($\bar{\text{x}}$ = 1.00 ± 0.072, *p* = 0.002) and NoT groups ($\bar{\text{x}}$ = 0.969 ± 0.047, *p* = 0.017) (Figure 6).

**FIGURE 7:**
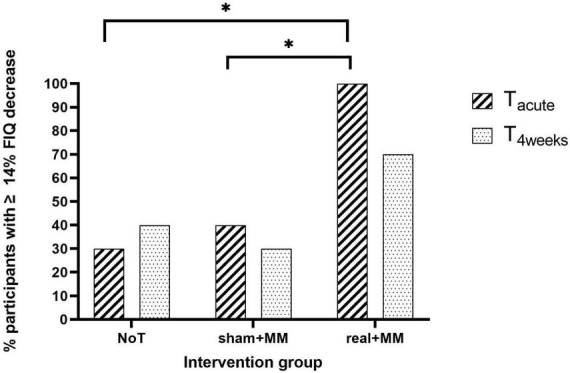
Clinically meaningful quality of life improvement. Bar chart illustrating the percentage of participants who reported ≥ 14% reduction in FIQ scores at T_acute_ and T_4weeks_ compared to baseline in the NoT, sham + MM and active + MM groups. * *p* < 0.05.

Regarding the clinical relevance of quality of life improvement at T_acute_ and T_4weeks_, we found a significant association between proportion of patients with ≥ 14% decrease in FIQ and group at T_acute_ (*p* = 0.003) but not at T_4weeks_ (*p* = 0.272). Bonferroni corrected pairwise comparisons between groups for patients with ≥ 14% FIQ reduction at T_acute_ showed that significantly lower number of patients in sham + MM and NoT groups demonstrated a clinically relevant improvement in quality of life than in active + MM (*p* < 0.05, Figure 7).

#### Pressure pain threshold

For the PPT, the MANOVA failed to show any significant difference among the three groups (F (1, 27) = 0.318, *p* = 0.73). For the PPT, no significant difference for the main interaction between TIME and GROUP was observed (F (4, 54) = 0.848, *p* = 0.501).

#### Sleep

The MANOVA to test whether the bimodal therapy was associated with improvement in sleep quality revealed no differences among groups (F_GG_ (2, 27) = 2.74, *p* = 0.083) and no significant GROUP-WEEK interaction (F_GG_ (8.56, 115.53) = 0.87, *p* = 0.553. Nevertheless, we found a large significant main effect of WEEK (F_GG_ (4.28, 115.53) = 4.78, *p* < 0.001, η^2^ = 0.150). *Post hoc* analysis showed the overall sleep quality of participants in the study (N = 30) was higher at PostStim4 than baseline (*p* = 0.003). Regarding sleep quantity, we did not find any significant results (GROUP: F_GG_ (2, 27) = 0.643, *p* = 0.534; WEEK: F_GG_ (4.01, 109.76) = 2.21, *p* = 0.072; interaction: F_GG_ (8.13, 109.76) = 1.33, *p* = 0.235).

#### Psychological wellbeing

**FIGURE 8:**
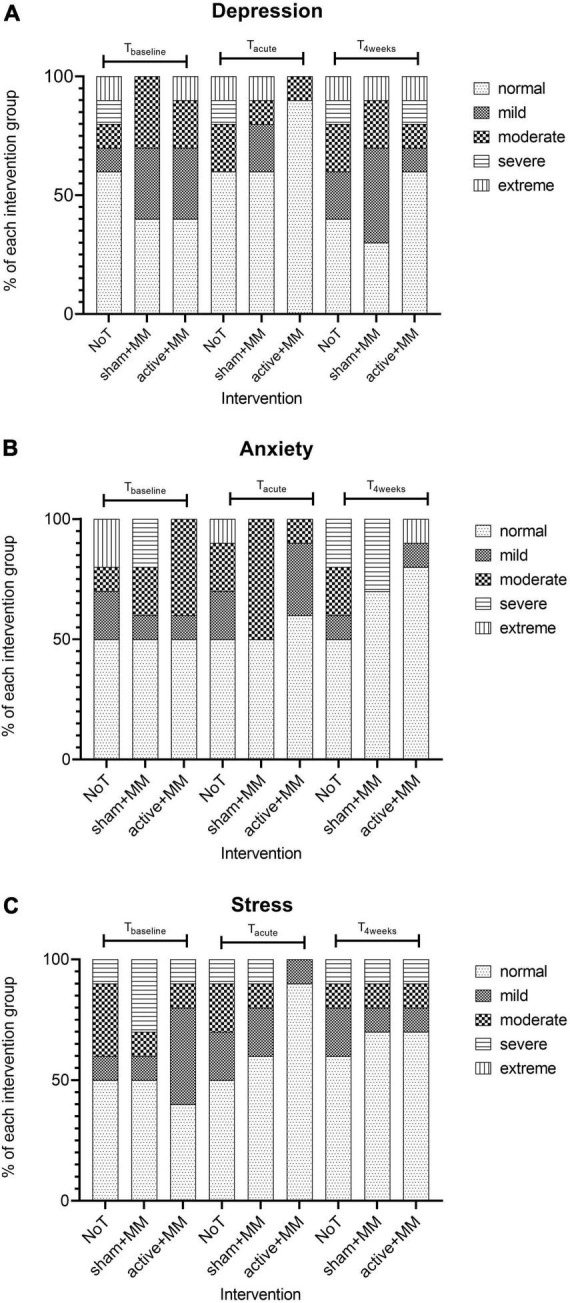
Effects of the tDCS and MM therapy on psychological wellbeing. Bar charts illustrating the changes in the percentage of participants with different levels of severity of **(A)** depression **(B)** anxiety and **(C)** stress in each intervention group at baseline, up to five days and 4 weeks after last stimulation session.

We did not find any significant association between symptom severity of each of the DASS subscales and group at T_baseline,_ T_acute_ and T_4weeks_ (*p* > 0.05). Based on the visual representation of the data (Figure 8), we can note the following observations: (1) for all the subscales, the percentage of patients with normal level is higher at T_acute_ and T_4weeks_ compared to baseline; (2) the percentage of patients with normal anxiety and depression level is higher in active + MM than in other groups at T_acute_ and T_4weeks_; (3) the percentage of patients with normal stress levels is higher in active + MM than in other groups only at T_acute_ but is equal between active + MM and sham + MM at T_4weeks_.

#### Assessment of the brief mindfulness meditation training

No significant differences were observed in mindfulness level as assessed by the 14-item FMI questionnaire after the week of MM training compared to before (F (1, 18) = 3.38, *p* = 0.082). No main effect of group (F (1, 18) = 0.297, *p* = 0.593) or significant interaction between group and time (F (1, 18) = 3.81, *p* = 0.067) was found.

Out of the 20 participants who underwent the one-week MM training, 85% (n = 17) reported that they felt that they truly meditated on each of the two last days of training. Only one patient reported not having the feeling of meditating on both days.

### Adverse effects

None of the participants discontinued the stimulation or required a medical intervention during or following stimulation. Table 3 sums up the adverse effects after 20 minutes of tDCS paired with meditation, including both sham and active stimulation conditions. Light headache was the most common adverse consequence; it was reported by 80% of participants after active tDCS paired with MM; however, also by 70% in the sham + MM group. Fatigue, being the second most common adverse effect, was reported by 70% of patients following anodal stimulation and 50% after sham stimulation. There were no significant difference in the occurrence of adverse effects between the two intervention groups. None of the participants reported long lasting adverse events of the MM and stimulation therapy.

**TABLE 3:** Adverse effects after concurrent stimulation and MM.

Adverse effects	Sham + MM (*n* = 10)	Active + MM (*n* = 10)	*P-*value
Uncomfortable feeling under electrodes	3 (30%)	3 (30%)	1.00
Light headache	7 (70%)	8 (80%)	1.00
Vertigo	4 (40%)	0 (0%)	0.65
Fatigue	5 (50%)	7 (70%)	0.87
Nervousness	3 (30%)	0 (0%)	0.21
Skin redness	3 (30%)	2 (20%)	1.00

Values are reported as n (%). Categorical variables were analyzed using Fisher-Freeman-Halton exact test.

## Discussion

The main objective of this proof-of-concept pilot clinical trial was to test the effectiveness and feasibility of ten daily sessions of 2 mA anodal tDCS of the left M1 paired with MM over two weeks (Monday to Friday) to reduce pain and to improve associated symptoms in FMS patients briefly trained in MM. We compared the effects of active tDCS during MM to sham tDCS during MM and NoT on NRS pain intensity (primary outcome), associated FMS symptoms (pain sensitivity, psychological impairment, sleep quality and sleep quantity) and quality of life of patients. Contrary to our hypothesis, the active + MM group did not show reduced clinical pain over time compared to sham + MM and the NoT groups. Patients in all three groups exhibited reduced pain intensity immediately after the two-weeks of intervention period compared to baseline indicating a non-specific effect. Among the secondary outcomes investigated in the study, we only found significantly higher quality of life in the active + MM group compared to the control groups. In a responder analysis, a larger number of FMS patients showed clinically meaningful improvement in FIQ scores in the active + MM group compared to the other groups immediately after treatment (T_acute_) but not at T_4weeks_.

### Efficacy of concurrent mindfulness meditation and transcranial direct current stimulation in fibromyalgia syndrome

Repetitive anodal tDCS over the left M1 received a level-B recommendation as probably effective in pain reduction in FMS patients (11, 19). Despite the elusive nature of mechanisms for the etiology and pathogenesis of FMS, increasing scientific evidence points out that central sensitization and impaired descending analgesic modulation contribute to the underlying mechanisms of this chronic pain condition. FMS patients have been previously shown to exhibit hyperactive thalamic function, associated with a diminished descending pain inhibition sustained by persistent excitatory nociceptive inputs (63). The analgesic effects of repetitive tDCS is believed to start with a potentiated excitability of the M1, which is sustained over time *via* long-term potentiation mechanisms (64, 65). This potentially leads to normalizing the hyperactivity of the thalamus through the modulation of antidromic inhibitory thalamic neurons, conducive to activation of the analgesic descending pain pathways (66–68). However, the exact underlying mechanisms are still unclear. The current intensity and the duration of a single stimulation session may significantly influence the influence of the tDCS protocol on the excitability of M1. Recently, increasing the intensity above 1 mA and increasing stimulation session durations above 26 min have been shown to reverse the excitability-enhancing effects of anodal tDCS on corticospinal excitability, pointing out the non-linear effects of tDCS (69, 70). However, none of the two studies investigated the aftereffects of tDCS at 2 mA delivered for 20 min. Jonker et al. (71) failed to show an effect of 2 mA anodal tDCS over left M1 for 20 min on cortical excitability, measured with transcranial magnetic stimulation (TMS) (71). It is important to note that the cited studies test healthy participants and the cortical excitability of FMS patients are healthy controls differ. Fibromyalgia patients have been shown to exhibit higher motor cortex excitability and less intracortical inhibition than healthy controls (72). Elevated intracortical inhibition has also been demonstrated following therapeutic interventions such as tDCS, repetitive TMS and aerobic exercise in FMS patients compared to sham groups (72). Further studies testing the effects of anodal tDCS intervention in FMS patients on M1 excitability is required to elucidate the exact underlying mechanisms of its analgesic benefits.

Moreover, investigating the neural mechanisms underlying mindfulness meditation-induced pain reduction using fMRI in an experimental pain induction task in healthy individuals, Zeidan et al. (73) found significant strong deactivations in both the right and left thalami (73). A recent study by Riegner et al. (74) demonstrated that pain relief during mindfulness meditation involves a pain modulatory mechanism mediated by a greater decoupling between the prefrontal cortex and the thalamus, which bypasses the traditional descending inhibitory pathways (75). The augmented thalamic deactivations due to combined MM and anodal tDCS effects to decrease pain perception in the patients formed the rationale behind combining the two interventions in our study.

Previous clinical studies testing the efficacy of pairing tDCS with MM or other non-pharmacological interventions demonstrated improvement in pain, quality of life and/or disease-related symptoms in chronic pain conditions, such as FMS, neuropathic pain and knee osteoarthritis among others. Combining MM and tDCS in knee osteoarthritis reduced pain intensity, pain sensitivity and disease-symptoms as well as enhanced conditional pain modulation in the active treatment group, compared to sham (32, 75). FMS patients receiving an intervention of active tDCS combined with concurrent aerobic exercise reported decreased pain intensity and anxiety levels compared to those in the sham group (76). Similar therapeutic effects have been observed by combining strengthening exercise with tDCS in knee osteoarthritis (77) or adding tDCS to mirror therapy in neuropathic pain patients (78). Despite the above evidence for larger improvement of symptoms in chronic pain during active tDCS than sham, we did not observe any larger reduction in pain intensity and associated symptoms in the active + MM group, compared to the sham + MM and NoT groups. One similar small study, combining tDCS (active vs sham) with a week of a multidisciplinary rehabilitation program (Riberto et al. (79)) failed to show pain relieving effects in fibromyalgia with a small improvement in quality of life measured by the 36-item Short Form Health Survey, which is in line with our current findings. In addition, despite the already cited evidence for a tDCS effect in FMS, a recent study with 15 sessions of anodal tDCS over three cortical targets in FMS showed neither pain relieving effects nor improved quality of life compared to sham (21, 80).

Concerning the pressure pain thresholds, our lack of significance among the groups is in line with most of previous studies failing to demonstrate an effect of tDCS on pain sensitivity in FMS (16, 21, 76). Whilst PPT might be a useful technique in distinguishing between healthy individuals and FMS patients (81) or to categorize patients in subgroups based on degree of tenderness and severity of psychological impairments (82), it might not be an accurate long-term measure for pain sensitivity or even therapeutic response in patients.

The higher quality of life observed in the active group, compared to the sham and NoT groups, despite the lack of other group effects might allow some speculation on other possible mechanisms involved in the combination of MM and tDCS in fibromyalgia patients. FMS patients exhibit a hyperactive sympathetic nervous system (83, 84). These dysfunctions result in diminished heart rate variability, which is linked to difficulty in emotional regulation (85, 86). Previous studies have also shown a strong negative correlation between quality of life and sympathetic activity in FMS (87, 88). Hence, combining meditation and tDCS in fibromyalgia might also affect an imbalance in the autonomic nervous system in patients with respective changes, rather than engaging the nociceptive pathways. Future studies with a more mechanistic focus would be needed to further investigate such non-nociceptive interactions.

### Brief mindfulness meditation training efficacy

This study used a one-week MM training prior to the intervention in a clinical cohort using a standardized training procedure. The MM training was chosen as a clinically feasible although limited training yet sufficient to establish a different and MM-based setting for the concurrent stimulation. Zeidan et al. (89) demonstrated that a three-day or a four-day 20 min daily MM practice showed enhanced mindfulness levels, as assessed by the FMI (73, 89, 90), however, in healthy participants exposed to experimental pain. In this study, we used the FMI as a tool to assess whether participants learned the technique of mindfulness practice. However, the mean FMI scores did not change after the training. Our findings possibly demonstrated that the 14-item FMI was not sensitive to five days of daily 25 min of MM practice in FMS patients. On the other hand, the attempt to quantify the construct of subjective mindfulness experience using self-report questionnaires faces many conceptual and methodological challenges in the field of contemplative sciences (91). In contrast, 85% of the patients who undertook the meditation training reported that they subjectively felt that they truly meditated on the last two days of the MM training, which supports a successful manipulation check for the brief MM training intervention.

### Strengths and limitations

Two strengths of our methodology are the inclusion of the NoT group and a MM training phase. The pain and symptoms experienced by FMS patients are variable, with fluctuating intensity over time and the course of the condition (92). This has been observed in this current study by the presence of participants in the NoT groups who showed clinically meaningful improvement in quality of life or pain intensity. Such fluctuations were also noticed in the percentage of participants with normal psychological scales. Therefore, including a no intervention control group allowed us to quantify any differences between sham and active groups compared to the NoT group.

An important limitation of published literature (32, 75) or ongoing studies (93) combining MM and tDCS in chronic pain remains the lack of a dedicated training phase for participants to learn and get familiarized with the technique of MM, as well as validated training protocols. To test the combinatory effects, studies should be designed to ensure that the patients are actually practicing meditation when receiving the adjunct tDCS intervention. In the case of the above-mentioned studies, it is difficult to relate the significance of their therapeutic effects to the consequence of ten days of combinatory therapy since it is impossible to know if the patients were able to successfully meditate during these ten days. Before this moment of ‘actual’ meditation, the observed analgesic effects could be due to only the anodal M1 stimulation. Our study design improves on this issue by including a week of standardized daily meditation training with feedback sessions prior to the concurrent MM and tDCS treatment. At the same time, the actual training procedure was based on clinical experience with meditation practice and not validated in previous studies.

The findings of this trial must be interpreted in the light of our study limitations. The underpowered sample size indeed hampers the quality of the efficacy investigated. No monotherapy groups were included in our clinical trial, making it impossible to elucidate how the effects of this bimodal intervention on clinical outcomes compare to tDCS only and MM only intervention effects. Since we were interested in proof of concept in a fairly typical clinical sample, patients were allowed to continue their medication and non-pharmacological treatment regimens throughout the study; however, participants were told not to change the dosage and type of medication or therapy. Participants were taking different types of medication in different doses including NSAIDs, tricyclic antidepressants, selective serotonin reuptake inhibitors, and selective norepinephrine reuptake inhibitors, owing to the heterogeneity of FMS symptoms. Medication withdrawal in FMS trials is a challenge for patients and has its own bias in “selecting” specific patients. Furthermore, the treatment was administered as a group therapy, which allowed for interaction between the participants either during or after the sessions. This could have led to additional non-specific psychological influences on patients, i.e., one participant complaining about their situation might negatively influence another, or positive group experiences reducing subjective symptoms. It is important to note the lack of age-matched groups in the study. However, a study comparing the differences in effects of anodal tDCS over M1 between young and elderly adults shows no significant differences in corticospinal excitability (94). Furthermore, according to a meta-analysis by Saldanha et al. (95), the analgesic effects following anodal tDCS over M1 compared to sham does not differ between elderly and younger patients (95). Moreover, despite a sex-matched design, both participating males were allocated in the sham group. It has been shown that male and female chronic pain patients perceive, modulate and respond to treatment differently (96, 97), and this discrepancy should be addressed in future studies.

Finally, the challenges associated with a clinical trial run during the global COVID-19 pandemic should not be overlooked. Strict hygiene guidelines were put forth during our experiments such as constant wearing of masks and distance between patients. Some participants reported discomfort and difficulty in breathing attributed to the wearing of masks during meditation. Other confounding factors associated with the pandemic, which could not be controlled for in this study, were subjects receiving COVID-19 vaccination, illness or demise of participants’ relatives as well as the fluctuating nature of the outbreak.

## Conclusion

Two weeks of an innovative bimodal intervention, concurrently combining tDCS and MM failed to improve clinical pain and associated symptoms in FMS patients briefly trained in meditation, without any serious adverse effects. All patients reported a non-specific decrease in pain intensity immediately after the two weeks of intervention compared to baseline and this decrease was observed in all groups, even the one not receiving any treatment. Still FMS patients in the active group reported clinically meaningful enhanced quality of life than those in the control groups immediately after the stimulation. The major limiting factor in this study was the underpowered sample size. Future research with larger samples and extended follow-ups is required to further test the efficacy as well as to unravel the potential mechanisms underlying therapeutic effects of a combined MM and tDCS therapy.

## Data availability statement

The raw data supporting the conclusions of this article will be made available by the authors, without undue reservation.

## Ethics statement

The studies involving human participants were reviewed and approved by Ethics Committee of the University Medical Center Göttingen. The patients/participants provided their written informed consent to participate in this study.

## Author contributions

PR: study design, writing the manuscript and proofreading. PR and SK: data collection and analysis. PR, SK, FP, and AA: data interpretation. FP and AA: supervising study design. AA: supervising clinical trial and data analysis. All authors contributed to the article and approved the submitted version.
